# Tissue-nonspecific alkaline phosphatase deficiency is associated with altered immune cell profiles in hypophosphatasia

**DOI:** 10.3389/fimmu.2026.1906557

**Published:** 2026-09-14

**Authors:** Luis Martínez-Heredia, Manuel Muñoz-Torres, Trinidad González-Cejudo, María Dolores López-Robles, Mónica Cabeo, Ángel Carazo-Gallego, Enrique García-Recio, Sheila González-Salvatierra, Victoria Contreras-Bolívar, María Carmen Andreo-López, Iván Iglesias-Baena, Francisco Andújar-Vera, Cristina García-Fontana, Beatriz García-Fontana

**Affiliations:** 1 Instituto de Investigación Biosanitaria de Granada (Ibs. Granada), Granada, Spain; 2 Biomedical Research Network in Fragility and Healthy Aging (CIBERFES), Instituto de Salud Carlos III, Madrid, Spain; 3 Endocrinology and Nutrition Unit, University Hospital Clínico San Cecilio, Granada, Spain; 4 Department of Medicine, University of Granada, Granada, Spain; 5 Clinical Analysis Unit, University Hospital Clínico San Cecilio, Granada, Spain; 6 Sensactive Technology, Granada, Spain; 7 Department of Nursing, Faculty of Health Sciences, University of Granada, Melilla, Spain; 8 Instituto de Investigación Biomédica de Málaga (IBIMA), Málaga, Spain; 9 Department of Endocrinology and Nutrition, Hospital Regional Universitario de Málaga, Málaga, Spain; 10 Bioinformatic Unit, Instituto de Investigación Biosanitaria de Granada (Ibs. Granada), Granada, Spain; 11 Department of Computer Science and Artificial Intelligence, Universidad de Granada, Granada, Spain; 12 Andalusian Research Institute in Data Science and Computational Intelligence (DaSCI Institute), Granada, Spain

**Keywords:** calprotectin, flow cytometry, hypophosphatasia, immune-mediated inflammatory diseases, TNSALP

## Abstract

**Background:**

Hypophosphatasia (HPP) is a rare metabolic disorder caused by mutations in the *ALPL* gene leading to tissue-nonspecific alkaline phosphatase (TNSALP) deficiency. Although traditionally considered a skeletal disease, emerging evidence suggests an immunomodulatory role for TNSALP in systemic inflammation and immune-mediated inflammatory diseases (IMIDs). This study investigated clinical, biochemical, and immunological features associated with inflammation in HPP.

**Methods:**

Forty-seven genetically confirmed HPP patients and 30 healthy controls with a comparable age and sex distribution were evaluated. Analyses included serum alkaline phosphatase (ALP), bone-specific ALP (BALP), C-reactive protein (CRP), fecal calprotectin (FCP), and flow cytometry in a subset of 29 HPP patients and 26 controls to assess leukocyte subpopulations and TNSALP expression, with confirmation by confocal microscopy.

**Results:**

HPP patients exhibited reduced TNSALP expression in T cells, B cells, neutrophils, and monocytes, together with lower CD3 and CD8 fluorescence intensity and an increased CD16+/CD14+ monocyte ratio. IMIDs were frequent in this referral cohort and were associated with lower ALP and higher FCP. Within the flow-cytometry subset, lower proportions of CD8+ cells within the CD3−/CD19− NK-enriched population and of TNSALP+ B cells were associated with IMID status.

**Discussion:**

These cross-sectional findings identify an association between TNSALP deficiency and a coordinated immune-inflammatory phenotype in HPP. They support the biological plausibility of an immunomodulatory role for TNSALP but do not establish that TNSALP deficiency causes inflammation or the development of IMIDs.

## 1 Introduction

Immune-mediated inflammatory diseases (IMIDs) are a group of chronic conditions characterized by impaired immune responses, leading to inflammation. These diseases can affect several tissues and organs and often share genetic and environmental risk factors (1). IMIDs constitute an important group of chronic disorders in the European population (2). Patients with inflammatory bowel disease (IBD) are at increased risk of developing other IMIDs, and common immune and inflammatory processes influence the coexistence of these conditions (3).

Regarding alkaline phosphatases, loss-of-function mutations in the *ALPI* gene, which encodes intestinal alkaline phosphatase (IAP), have been associated with inflammatory bowel disease in humans (4). IAP and tissue-nonspecific alkaline phosphatase (TNSALP) are distinct isoforms encoded by different genes; therefore, evidence related to ALPI/IAP should not be interpreted as evidence that *ALPL* variants or reduced TNSALP activity cause intestinal inflammation. Rather, these observations provide broader biological context for a potential role of alkaline phosphatases in intestinal immune homeostasis. TNSALP has independently been linked to immune regulation (5), including dephosphorylation of pathogen-associated and damage-associated molecular patterns such as bacterial lipopolysaccharide (6) and extracellular ATP (7). TNSALP expression has been described in neutrophils (8) and macrophages (9), and murine studies have implicated it in lymphocyte activation (10). However, the relationship between TNSALP deficiency and immune-mediated inflammatory diseases in HPP has not been directly established.

Loss-of-function mutations in the *ALPL* gene, which encodes TNSALP, lead to hypophosphatasia (HPP), a condition characterized mainly by impaired bone mineralization and recurrent fractures (11). HPP is considered a rare disease, with a prevalence estimated at approximately 1/2430 individuals in Europe (12) and 1/1692 individuals in the Spanish population (13). Due to its broad clinical spectrum, HPP is frequently misdiagnosed as more prevalent conditions such as osteoporosis (14). Our previous observational studies reported a high frequency of IMIDs among patients with HPP (13), which motivated the present investigation. We therefore hypothesized that reduced ALP activity associated with *ALPL* variants may also be associated with immune-inflammatory alterations in HPP.

## 2 Materials and methods

### 2.1 Study design

This descriptive study compared a cohort of healthy controls with a cohort of patients with HPP. The HPP cohort was subsequently stratified according to the presence or absence of clinically diagnosed IMIDs and according to elevated FCP status.

### 2.2 Study population

In this cross-sectional study, 47 adult patients diagnosed with HPP (40.5% males, 59.5% females), aged 18 to 80 years, were included. For subgroup analyses, patients were categorized according to the presence or absence of clinically diagnosed immune-mediated inflammatory diseases (IMIDs) and according to FCP status. IMIDs comprised chronic inflammatory disorders of immune-mediated origin previously diagnosed by the corresponding specialists. FCP levels >50 µg/g were used to define elevated FCP, as a surrogate of increased intestinal inflammatory activity rather than as a diagnosis of a specific gastrointestinal disorder. HPP patients were recruited from the Endocrinology and Nutrition Unit at the University Hospital Clinico San Cecilio of Granada (Spain) between 2021 and 2024. Our unit receives referrals for HPP assessment from other provinces and regions of Spain, although most participants in the present cohort were recruited locally in Granada. The inclusion criteria required persistently low serum ALP after excluding secondary hypophosphatasemia and treatments or diseases known to lower ALP levels (15). Individuals with persistently low ALP of unknown cause were contacted and invited to participate in the study, which involved an interview, physical examination, and collection of stool and blood samples for biochemical analysis and genetic screening. In addition, 30 healthy individuals with a comparable age and sex distribution were included as controls. Healthy controls had no history of fractures unrelated to high-impact trauma or premature tooth loss and had no known gastrointestinal disease or relevant gastrointestinal symptoms at inclusion. No participant had evidence of an acute infection at the time of study assessment. Before participation, written informed consent was obtained from each subject. The study was conducted in accordance with the Declaration of Helsinki and was approved by the Research Ethics Committee of Granada (Project ID: 0885-N-21; June 14, 2021).

### 2.3 Clinical evaluation and biochemical analysis

The height and weight were measured at the baseline according to standard procedures. The body mass index (BMI) was calculated by the Quetelet formula, weight (kg)/stature (m^2^).

For biochemical measurements, venous blood samples were collected in the morning after fasting overnight, and serum samples were stored at -80 °C until analysis. Complete blood tests were taken from the patients, including red blood cells (RBCs) count, platelet count, lymphocyte, monocyte, neutrophil, eosinophil, and basophil percentages. Parameters such as C-reactive protein (CRP), iron, ferritin and transferrin saturation index (TSI) were measured using automated clinical chemistry analyzers following standardized enzymatic and immunoturbidimetric methods in the hospital laboratory. The alkaline phosphatase (ALP) levels were measured by a colorimetric method in an AU5800 analyzer (Beckman Coulter). Bone isoform of alkaline phosphatase (BALP) was also measured using an immunoenzymatic assay in an AU5800 analyzer (Beckman Coulter). For each patient, three routine measurements of ALP and BALP obtained at different time points were averaged for analysis. Repeated measurements were used to confirm consistently reduced ALP and BALP levels and thus support the biochemical characterization of HPP.

Fecal calprotectin (FCP) was measured by particle-enhanced turbidimetric immunoassay (PETIA) using the BÜHLMANN fCAL turbo reagent (B-KCAL-RSET; BÜHLMANN Laboratories AG) on an Abbott Alinity clinical chemistry analyzer (Abbott Laboratories).

### 2.4 Cell staining, flow cytometry and confocal microscopy

Peripheral blood samples were collected in lithium heparin tubes from 29 HPP patients and 26 control participants for flow cytometry analyses. Afterward, 200 µL of each sample was incubated with 100 µL of phosphate-buffered saline (PBS 1X), 5 µL of Fc blocker (BD Biosciences), and the corresponding antibody cocktail (**Supplementary Tables 1**, **S2**) for 20 minutes. The lymphoid panel identified CD3+ T cells, subdivided into CD4+, CD8+, double-negative (DN), and double-positive (DP) subsets; B cells as CD3-/CD19+; and CD3-/CD19- lymphocytes within the lymphocyte gate as an NK-enriched population. This gating approach is supported by published data showing that 93-96% of sorted CD3-/CD19- lymphocytes expressed canonical CD56/CD16 NK markers (16). Because CD56 and CD16 were not included in our lymphoid panel, these cells are referred to throughout as an NK-enriched population and were further stratified according to CD8 expression without inferring cytotoxic function. The myeloid panel included neutrophils (CD66b+/CD16+), eosinophils (CD66b+/CD16-), and monocytes subdivided into classical (CD14+/CD16-), intermediate (CD14+/CD16+), and non-classical (CD14-/CD16+) subsets. Antibodies, fluorochromes, and clones are detailed in **Supplementary Tables 1** and **S2**.

Red blood cells were lysed and the leukocytes fixed using RBC Lysis/Fixation Solution 1X (Biolegend) for 20 minutes at room temperature. The suspension was then centrifuged for 10 minutes at 4000 rpm and the supernatant was discarded. The cells were washed twice with RBC Lysis/Fixation Solution (10X) and centrifuged for 10 minutes at 4000 rpm each time. Finally, the supernatant was removed, and the white blood cells (WBCs) were resuspended in 300 µL of PBS 1X. Isotype control was used for TNSALP determination. The results were obtained using the Cytek™ Northern Lights Flow Cytometry System cytometer and analyzed with FlowJo V10.9.

Healthy donor samples were stained with APC anti-TNSALP and lineage markers for T cells (CD3), B cells (CD19), monocytes (CD14), and neutrophils (CD16). Sample processing, including red blood cell lysis, leukocyte fixation, washing steps, and resuspension in PBS 1X, was performed as described above, and isotype-matched controls were used to determine TNSALP-specific staining.

The different leukocyte populations were subsequently sorted using a BD FACS Melody cell sorter. Neutrophils were isolated based on PE anti-CD16 expression and side scatter properties. Sorted cells were then stained with Hoechst 33342 nuclear dye and finally captured using a ZEISS LSM 900 confocal microscope equipped with a 63× objective. Images were processed with ImageJ software.

### 2.5 Statistical analysis

Analyses and figures were performed in RStudio. Differences in binomial variables, such as sex, treatment with non-steroidal anti-inflammatory drugs (NSAIDs), or glucocorticoid therapy, were assessed using Fisher’s exact test, while continuous variables were compared using the Mann-Whitney U test. Exploratory nonlinear associations were assessed using quadratic polynomial regression, and correlations were evaluated using Spearman coefficients. Statistical significance for the primary comparisons was set at *p* < 0.05. Regression analyses were exploratory and were selected according to the data structure and available sample size. Bayesian logistic regression was used to examine associations of clinical variables with IMID status. LASSO logistic regression with 5-fold cross-validation was used for variable selection and penalty tuning in analyses of elevated FCP status. Standard binary logistic regression was used for the limited set of flow-cytometry variables. A threshold of *p* < 0.10 was used only to screen potentially relevant associations or candidate variables in exploratory modeling; associations with *p* ≥ 0.05 were not considered conventionally statistically significant. ROC curves and AUCs were used to describe apparent discrimination within the study sample and should not be interpreted as externally validated predictive performance. For exploratory logistic regression and LASSO models, potential optimism in model discrimination was assessed using 1, 000 bootstrap resamples. In each bootstrap replicate, the model was refitted and the AUC was calculated both in the bootstrap sample and when applied to the original dataset. Optimism was defined as the difference between these two AUC estimates, and the mean optimism across bootstrap replicates was subtracted from the apparent AUC to obtain an optimism-corrected estimate. Given the exploratory nature and limited sample size of these analyses, the resulting estimates were considered hypothesis-generating rather than validated measures of predictive performance.

## 3 Results

### 3.1 Clinical outcomes in HPP patients

Mutations in the *ALPL* gene were identified in all included patients (28 females and 19 males, 59.5% and 40.5%, respectively). A total of 31.9% of patients (15/47) received NSAID treatment, while 10.6% (5/47) were receiving glucocorticoid therapy. None of the patients was receiving enzyme replacement therapy with asfotase alfa at the time of study assessment. Among those with available biochemical data, 71.8% (28/39) showed BALP levels below the normal range and 63.6% (21/33) had FCP levels >50 µg/g. For comparison, FCP levels >50 µg/g were observed in 11/30 (36.7%) healthy controls. Regarding clinical manifestations, IMIDs were present in 46.8% of patients (22/47). Fractures were observed in 42.6% (20/47), and tooth loss was reported in 61.7% (29/47). Patient characteristics and outcomes are detailed in **Supplementary Table 3**.

### 3.2 Clinical and biochemical characteristics of HPP patients

Clinical comparisons between healthy controls and HPP patients revealed significant differences in several biochemical and hematological parameters. HPP patients showed markedly lower ALP activity (*p* < 0.001) and BALP levels (*p* < 0.001), together with higher CRP (*p* = 0.005), FCP (*p* = 0.016), and circulating monocyte percentages (*p* = 0.014) (**Figures 1A–E**). In an exploratory analysis performed across the combined HPP-control sample, quadratic regression identified significant parabolic associations between ALP activity and log10-transformed FCP and CRP (**Figures 1F, G**; **Supplementary Tables 4**, **S5**). Because HPP patients and controls occupy different portions of the ALP range, these combined-cohort curves should not be interpreted as demonstrating an HPP-specific U-shaped relationship.

**Figure 1:**
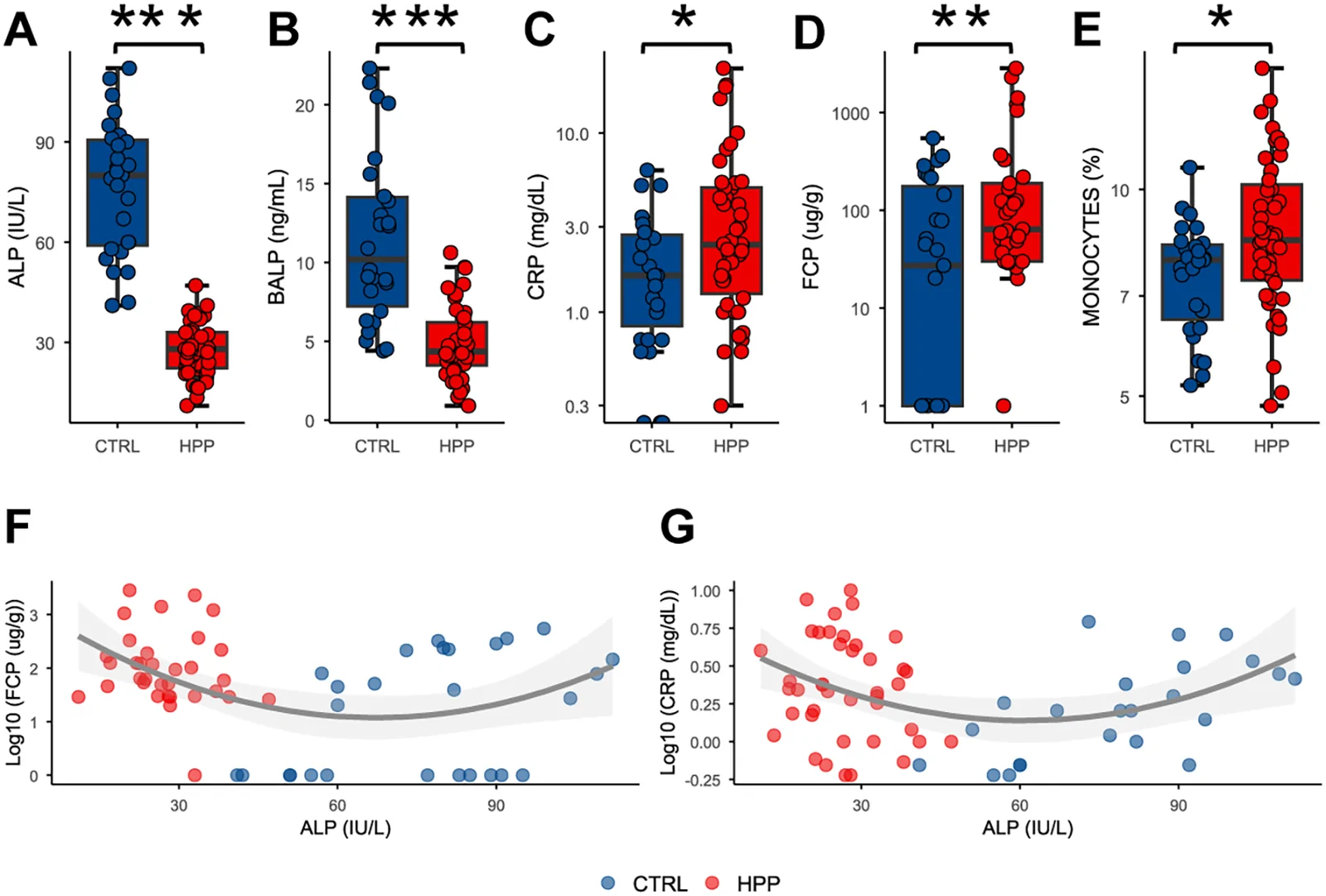
Boxplots of the biochemical variables that differed significantly between healthy individuals and HPP patients showing the median and interquartile range (IQR) **(A–E)**. Quadratic polynomial regressions of ALP activity against the log10-transformed FCP **(F)** and CRP **(G)**. (*) *p* < 0.05, (**) *p* < 0.01, (***) *p* < 0.001. ALP, alkaline phosphatase activity; BALP, bone specific alkaline phosphatase isoform; CRP, C-reactive protein; FCP, fecal calprotectin.

### 3.3 Immunophenotypic alterations in HPP patients and their relationship with bone and inflammatory biomarkers

Two multiparametric flow cytometry panels (**Supplementary Figures 1**, **S2**) were used to analyze leukocyte subpopulations and surface TNSALP expression in whole blood from controls and patients, with validation by confocal microscopy in T cells, B cells, neutrophils, and monocytes (**Figure 2A**). In UMAP representations, the highest TNSALP expression among lymphocytes was observed in T and B cells, whereas within the myeloid lineage it was mainly observed in neutrophils and eosinophils (**Figures 2B, C**). HPP patients showed a reduced percentage of TNSALP-expressing cells in CD4+, CD8+, DN, and DP T cells and B lymphocytes (p < 0.001) compared with controls (**Figure 2D**). CD8- and CD8+ subsets within the CD3-/CD19- NK-enriched population were unchanged. In the myeloid panel, HPP patients showed reduced proportions of TNSALP-expressing neutrophils (*p* < 0.001), eosinophils (*p* < 0.001), and monocytes (*p* = 0.006) (**Figure 2E**). HPP patients *also* exhibited a higher monocyte percentage (*p* = 0.019), an increased CD16+/CD14+ monocyte ratio (*p* = 0.007), and lower CD3 and CD8 median fluorescence intensity (MFI) in total T cells and CD8+ T cells (*p* < 0.001 and *p* = 0.034, respectively) (**Figure 2F**). These MFI differences are reported as phenotypic alterations and do not, by themselves, establish activation, exhaustion, or functional impairment.

**Figure 2:**
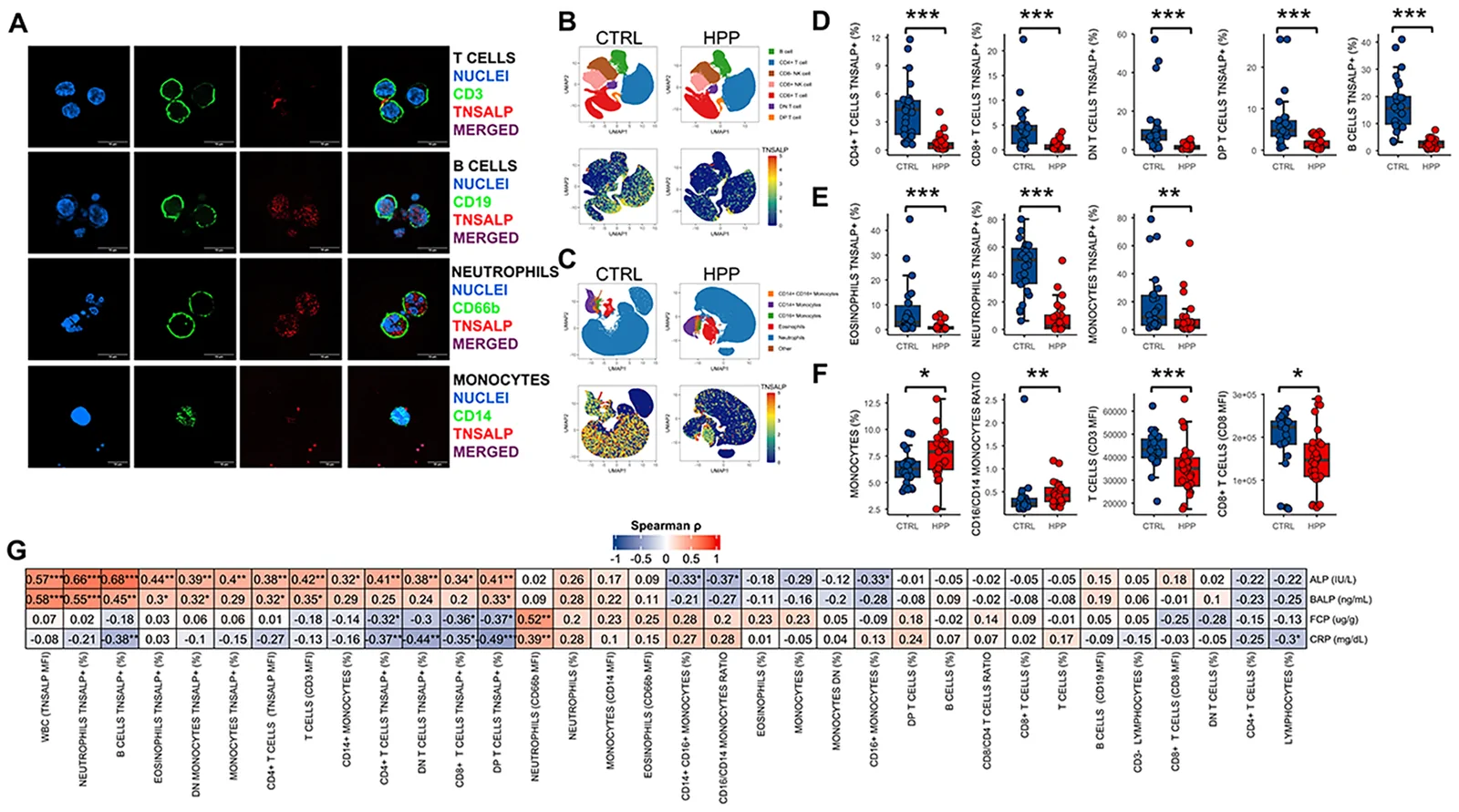
Confocal microscopy of TNSALP expression in T cells, B cells, neutrophils, and monocytes sorted using differential membrane markers for each population **(A)**. UMAP representations of lymphocyte subpopulations and TNSALP expression between HPP and control group **(B)**. UMAP representations of myeloid subpopulations and TNSALP expression among the study groups **(C)**. Boxplots of lymphoid panel subpopulations showing significant differences in TNSALP expression among the study groups showing the median and IQR **(D)**. Boxplots of myeloid panel subpopulations display showing TNSALP expression among the study groups showing the median and IQR **(E)**. Monocyte percentage, CD16/CD14 ratio, CD3 MFI in total T cells and CD8 MFI in CD8^+^ T cells showing the median and IQR **(F)**. Spearman correlations of ALP activity, BALP, FCP, and CRP with flow cytometry variables **(G)**. (*) *p* < 0.05, (**) *p* < 0.01, (***) *p* < 0.001. ALP, alkaline phosphatase activity; BALP, bone specific alkaline phosphatase isoform; CRP, c-reactive protein; FCP, fecal calprotectin; MFI, median fluorescence intensity.

Spearman correlation analysis between cytometric parameters and clinical biomarkers (ALP, BALP, FCP, and CRP) showed that ALP activity correlated positively with surface TNSALP expression across all leukocyte subpopulations and with CD3 antigen density, and negatively with the CD16^+^/CD14^+^ monocyte ratio. BALP levels also correlated positively with the percentage of TNSALP^+^ neutrophils, monocytes, B lymphocytes, and DN T cells, as well as with CD3 antigen density, and negatively with the percentage of CD16^+^ monocytes. In contrast, inflammatory biomarkers showed inverse associations with TNSALP expression in lymphocytes: FCP correlated negatively with TNSALP^+^ CD4^+^, CD8^+^, and DN T cells, while CRP correlated negatively with all lymphoid subsets analyzed (CD4^+^, CD8^+^, DN, and DP T cells). Additionally, both FCP and CRP correlated positively with CD66b antigen density in neutrophils, and CRP also correlated positively with the CD16^+^/CD14^+^ monocyte ratio (**Figure 2G**).

### 3.4 Clinical and biochemical profiles associated with IMIDs in HPP patients

HPP patients were stratified according to the presence of clinically diagnosed IMIDs. Those with IMIDs showed significantly lower ALP activity and RBC counts, and higher FCP levels compared with patients without IMIDs (**Figure 3A**), while no differences were observed in sex distribution or the use of NSAIDs and glucocorticoids (**Figure 3B**).

**Figure 3:**
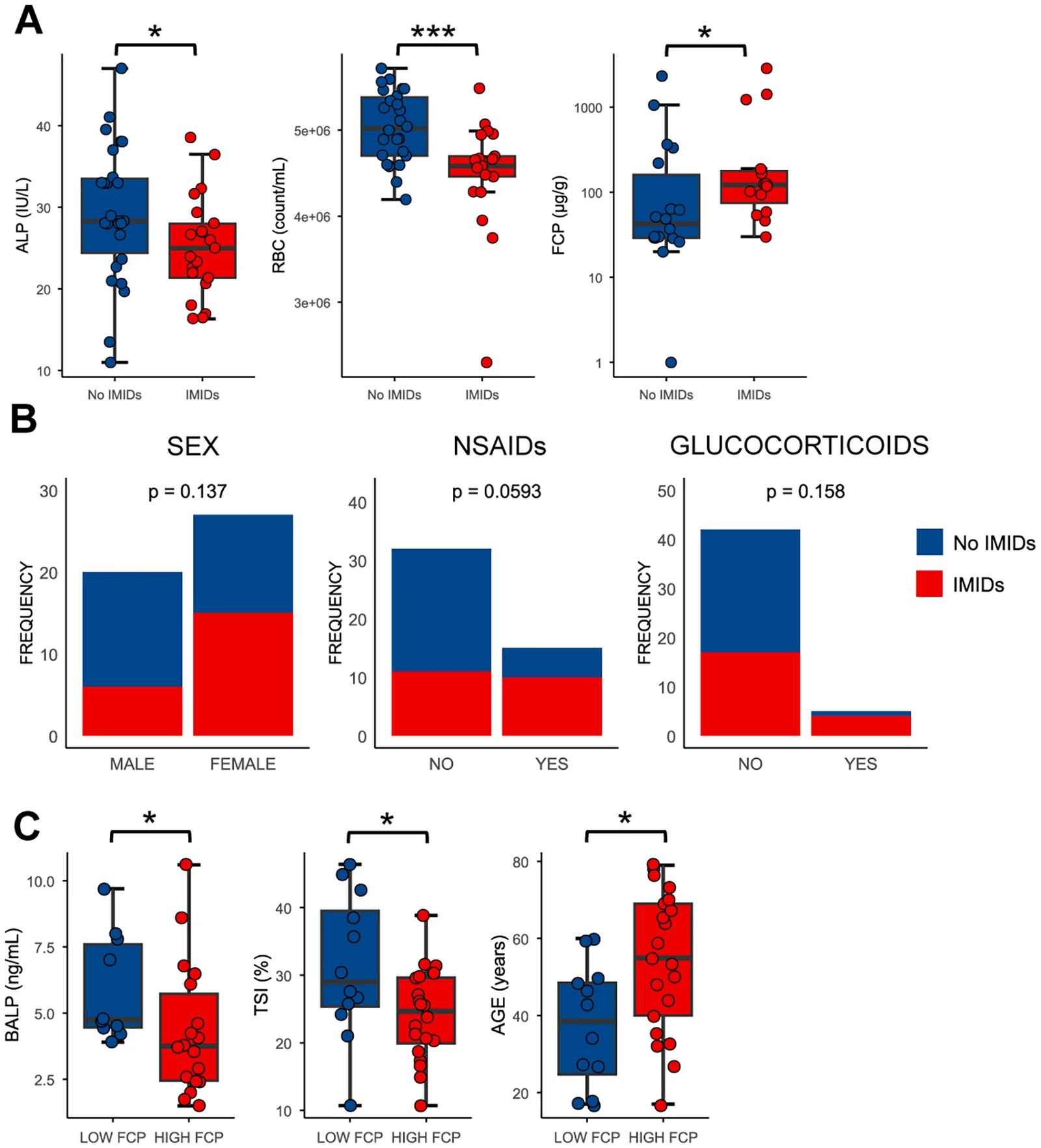
Boxplots of clinical variables showing significant differences between HPP patients with and without diagnosed IMIDs, showing the median and IQR **(A)**. Fisher’s exact test results for binomial variables (sex, NSAIDs, and glucocorticoid therapy) **(B)**. Boxplots of clinical variables with significant differences between patients with normal and elevated FCP according to the 50 µg/g threshold, showing the median and IQR **(C)**. (*) *p* < 0.05, (***) *p* < 0.001. ALP, alkaline phosphatase activity; RBC, red blood cells; FCP, fecal calprotectin; BALP, bone-specific alkaline phosphatase isoform; TSI, transferrin saturation index.

In exploratory Bayesian logistic regression analyses, lower ALP activity and higher FCP levels were associated with IMID status. Detailed regression estimates and apparent discrimination metrics are provided in the **Supplementary Material** (**Supplementary Tables 6–8**).

Finally, elevated FCP status (FCP >50 µg/g) was associated with lower BALP and TSI values and older age (**Figure 3C**). In an exploratory LASSO analysis, BALP, TSI, and age were retained after penalization. Detailed model estimates and apparent discrimination metrics are provided in the **Supplementary Material** (**Supplementary Table 9**).

### 3.5 Immunophenotypic features associated with IMID status in HPP

HPP patients with IMIDs showed a lower proportion of CD8+ cells within the CD3-/CD19- NK-enriched population (*p* = 0.0308) and a lower proportion of TNSALP+ B cells (*p* = 0.0305) (**Figures 4A, B**).

**Figure 4:**
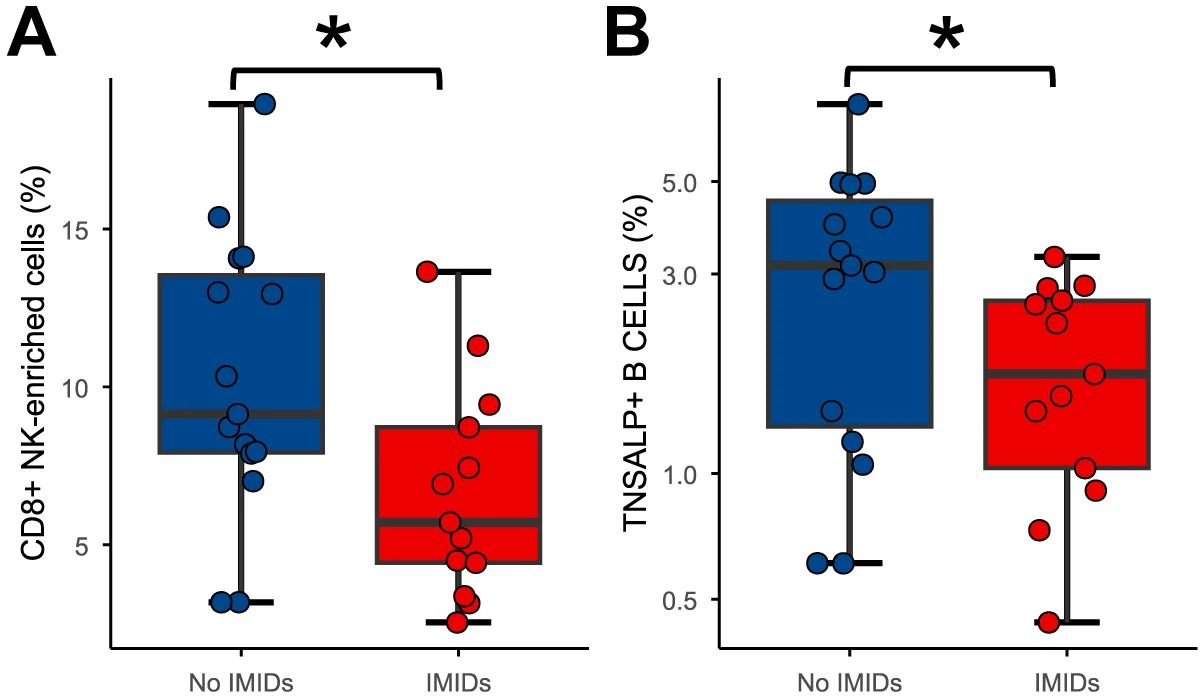
Percentage of CD8+ cells within the CD3-/CD19- NK-enriched population **(A)** and TNSALP+ B cells **(B)** in HPP patients with and without diagnosed IMIDs, showing the median and IQR. *p < 0.05.

In exploratory logistic regression analyses, lower proportions of CD8^+^ cells within the NK-enriched population and TNSALP^+^ B cells were associated with IMID status. Detailed regression estimates and apparent discrimination metrics are provided in the **Supplementary Material** (**Supplementary Tables 10**, **S11**).

In a multivariable exploratory logistic regression model including TNSALP^+^ B cells, CD8^+^ cells within the NK-enriched population, age, and sex, both immunophenotypic variables remained associated with IMID status. Detailed model estimates and apparent discrimination metrics are provided in the **Supplementary Material** (**Supplementary Table 12**).

## 4 Discussion

To our knowledge, this study provides one of the first integrated clinical, biochemical, and immunophenotypic characterizations of immune-inflammatory alterations in HPP. In our referral-based cohort, IMIDs were present in 46.8% of patients. Given the specialized recruitment setting and the potential for referral and ascertainment bias, this frequency should not be interpreted as a population prevalence estimate or directly compared with estimates from the general population. This observation is also consistent with previous reports describing HPP patients with systemic lupus erythematosus (17), psoriasis (18) and other inflammatory skin lesions (19), arthritis (20), ankylosing spondylitis (21), and chronic multifocal non-bacterial osteomyelitis (22). Altogether, these findings support a broader view of HPP as a systemic disorder in which impaired TNSALP activity may be associated not only with defective mineralization but also with immune dysregulation.

In line with this, HPP patients showed the expected reduction in circulating ALP activity together with lower BALP levels, but these abnormalities were also accompanied by increased CRP and FCP concentrations and a higher proportion of circulating monocytes. FCP levels >50 µg/g were observed in nearly two-thirds of the evaluated patients, indicating that elevated FCP was frequent in this subset; however, FCP is a nonspecific marker and this finding does not by itself establish subclinical intestinal disease. The exploratory quadratic associations observed between ALP activity and CRP/FCP in the combined HPP-control sample provided an additional characterization of the relationship between ALP activity and inflammatory markers. This exploratory analysis was intended to characterize the behavior of inflammatory markers across the full ALP range and should not be interpreted as demonstrating a disease-specific U-shaped relationship within HPP. One possible explanation is that increased ALP activity may reflect a compensatory response to inflammation, whereas markedly reduced ALP activity in HPP may coexist with persistent inflammatory signaling; this mechanistic interpretation remains hypothetical. Although RBC and iron-related changes were also observed in specific subgroups, particularly in HPP patients with IMIDs and in those with elevated FCP, these findings are better interpreted as supportive features of the inflammatory phenotype in HPP than as central mechanistic consequences of TNSALP deficiency (23, 24).

The immunophenotypic findings provide a potential cellular basis for this inflammatory background. We observed a generalized reduction in surface TNSALP expression across multiple leukocyte subpopulations, including neutrophils, monocytes, and B and T lymphocytes in HPP patients compared to controls. TNSALP expression has previously been documented in neutrophils (25), B cells (26), and phagocytes (9), whereas its presence in T cells remains controversial, with discrepancies between human (27) and murine (10) studies. In this context, the confocal microscopy data presented here provides, to our knowledge, the first evidence in human cells supporting TNSALP expression in T lymphocytes, while also visually confirming its expression in neutrophils, B cells, and monocytes. These data suggest that *ALPL* deficiency in HPP is not restricted to mineralizing tissues but also affects the immune compartment. As expected in the setting of *ALPL* deficiency, however, reduced TNSALP staining itself should not be interpreted as evidence of proinflammatory immune activation. Furthermore, the positive correlations between total ALP activity and the proportion of TNSALP^+^ leukocyte subsets support the biological coherence of the cytometric findings and raise the possibility that flow-cytometric assessment of TNSALP could serve as a complementary diagnostic approach in HPP. Agreeing, we registered an intellectual property (P202530172) based on TNSALP detection in neutrophils, the predominant WBC population in peripheral blood, for use in patients with suggestive clinical manifestations but inconclusive biochemical or genetic results. Although this strategy still requires external validation, it may help improve recognition of this underdiagnosed disease.

Beyond its potential diagnostic value, the immunophenotypic profile identified in our cohort is consistent with an altered immune-inflammatory phenotype in HPP. Reduced CD3 MFI in total T cells and lower CD8 MFI within CD8^+^ T cells have been described in contexts of persistent immune stimulation (28–31); however, these fluorescence-intensity differences alone cannot establish T-cell activation, exhaustion, or impaired cytotoxicity. In our patients, these alterations are particularly relevant because they occur in parallel with increased systemic and intestinal inflammatory markers, including CRP and FCP, as well as a high frequency of IMIDs, suggesting that these alterations may form part of the broader immune-inflammatory phenotype observed in HPP rather than representing isolated lymphocyte abnormalities. This interpretation is further supported by the innate immune compartment. The increased CD16/CD14 ratio observed in monocytes points to a shift toward a proinflammatory phenotype enriched in intermediate (CD14^+^CD16^+^) and non-classical (CD14^-^CD16^+^) subsets (32), populations that have been repeatedly linked to IMIDs such as rheumatoid arthritis (33) and Crohn’s disease (34), as well as to other chronic inflammatory conditions including cancer (35) and viral infections (36). Notably, this finding is consistent with the increased circulating monocyte counts observed in our HPP cohort, as detected both in routine clinical analyses and by flow cytometry, further supporting the presence of a sustained inflammatory state. The inverse associations of ALP and BALP with the CD16^+^ monocyte phenotype and CD16/CD14 ratio, together with their positive relationship with CD3 antigen density, reinforce the association between lower ALP activity and an altered immune-inflammatory profile. Similarly, the inverse correlations of CRP and FCP with TNSALP^+^ lymphocyte subsets, and their positive association with CD66b expression on neutrophils, connect these cellular alterations with clinically relevant inflammatory signals. Since CD66b is upregulated in activated neutrophils during inflammatory conditions such as infections (37) and is associated with enhanced adhesion and oxidative burst (38), these findings are consistent with an association between TNSALP deficiency and an altered systemic inflammatory environment, but they do not establish a specific functional immune mechanism.

A potential mechanistic hypothesis may involve impaired clearance of DAMPs. TNSALP has been implicated in the dephosphorylation and detoxification of extracellular nucleotides, and its deficiency could favor the accumulation of proinflammatory signals in the extracellular milieu (8). In this context, DAMPs such as high-mobility group box 1 (HMGB1) and cell-free DNA (cfDNA), including that released during neutrophil extracellular trap (NET) formation, have been associated with the severity of inflammatory conditions such as sepsis (41, 42), a state in which TNSALP is thought to exert a protective role by contributing to the detoxification of proinflammatory extracellular signals and limiting excessive inflammation (6). Moreover, DAMPs are known to contribute to the initiation and perpetuation of autoimmune responses, as illustrated in multiple sclerosis models where HMGB1 and heat shock protein 70 (HSP70) promote autoimmune neuroinflammation (39–41). This pathway was not directly tested in the present study and should therefore be considered a biologically plausible hypothesis requiring dedicated functional investigation.

In the subgroup of HPP patients with clinically diagnosed IMIDs, we observed higher percentages of total T cells, a lower proportion of CD8^+^ cells within the CD3^-^/CD19^-^ NK-enriched population, and a reduced percentage of TNSALP^+^ B cells. Because canonical NK-cell markers such as CD56 and CD16 and functional assays were not included in the lymphoid panel, CD8 expression within this NK-enriched gate cannot be used to infer cytotoxic capacity, exhaustion, or impaired surveillance. Particularly noteworthy was the decrease in TNSALP^+^ B cells, as previous studies have shown that TNSALP is expressed by CD27^-^ B lymphocytes (26) and increases upon B-cell activation (42), while ALP activity peaks after the proliferative phase (43), suggesting a closer relationship with activation status than proliferation itself. Therefore, the reduced proportion of TNSALP^+^ B cells in HPP patients with IMIDs may reflect disturbed B-cell activation dynamics or shifts in B-cell subset distribution within a chronically inflamed setting. However, reduced TNSALP staining is also expected in the context of *ALPL* deficiency and does not by itself constitute evidence of immune activation. The associations involving the NK-enriched CD8^+^ subset and TNSALP^+^ B cells should therefore be considered exploratory immunophenotypic findings that require functional and external validation.

Beyond the immunophenotypic profile, lower ALP activity and higher FCP levels were associated with IMID status. Exploratory model-based analyses are reported in the **Supplementary Material** and should be considered hypothesis-generating rather than validated predictive models.

When focusing specifically on elevated FCP status, defined according to the established FCP cut-off (>50 µg/g), HPP patients with elevated FCP were older and showed features compatible with inflammatory anemia, including lower transferrin saturation. Although total ALP activity did not differ significantly between these subgroups, BALP levels were notably lower in patients with elevated FCP. Exploratory model-based analyses of these associations are reported in **Supplementary Material** and should be considered hypothesis-generating rather than validated predictive models.

This study has several limitations, including modest sample size (47 HPP patients and 30 matched controls) and limited availability of FCP (33 HPP patients) and flow cytometry (29 HPP patients), which may reduce the statistical power and generalizability. Furthermore, although our center receives patients from different provinces and regions, the cohort remains referral-based and may therefore be subject to referral and ascertainment bias, which should be considered when interpreting the findings and their generalizability. Accordingly, the observed frequency of IMIDs cannot be used to estimate population prevalence or relative risk. The characterization of the IMID subgroup was also limited by the availability of detailed information on disease duration and disease activity, which was not systematically recorded for all patients. As IMID activity and treatment may influence inflammatory markers and immune phenotypes, this may have contributed to variability within the subgroup. Although FCP is an established biomarker of intestinal inflammation, elevated levels may occur in different gastrointestinal conditions and are not specific to a particular underlying cause. FCP may also be influenced by age, NSAID exposure, infection, and gastrointestinal comorbidity; although age and NSAID use were recorded and no acute infection was present at study assessment, detailed gastrointestinal comorbidity data were not systematically available for all participants. The discrimination estimates reported in the **Supplementary Material** reflect performance within the study cohort and may be subject to optimism given the limited sample size, particularly for the flow cytometry-based models. Therefore, these models should be considered exploratory and require external validation in larger, independent cohorts to confirm their discriminatory performance and generalizability. No formal calibration analysis or correction for multiple testing was performed, and the 5-fold cross-validation used in LASSO served for penalty selection rather than unbiased model validation. Additionally, immunophenotyping was restricted to a basic lymphoid and myeloid panel focused mainly on TNSALP expression, which limited the depth of immune characterization. Canonical NK markers such as CD56 and CD16 were not included in the lymphoid panel, and functional immune assays were not performed; consequently, CD3/CD8 fluorescence differences and the NK-enriched CD8^+^ subset cannot establish activation, exhaustion, or cytotoxic impairment. Future studies should therefore incorporate approaches to define specific T-cell subsets and dedicated functional assays to clarify the mechanisms linking TNSALP deficiency to immune dysregulation and chronic inflammation in HPP. Although all patients were genetically characterized, variant-specific residual enzyme activity was not experimentally measured and no formal genotype-phenotype analysis was attempted because of the limited sample size and marked genetic and clinical heterogeneity. Nevertheless, these limitations must be considered in the context of HPP as a rare and underdiagnosed disease, in which the recruitment of large, well-characterized cohorts remains particularly challenging, especially when genetic confirmation, clinical stratification, and extensive immunophenotyping are required. Despite this, our study has several important strengths. To our knowledge, this is one of the first studies to comprehensively explore the relationship between TNSALP deficiency and immune-inflammatory alterations in genetically characterized adult HPP patients by integrating clinical, biochemical, inflammatory, and flow cytometry-based immune profiling. Additionally, the internal stratification of the HPP cohort according to clinically diagnosed IMIDs and elevated FCP status allowed a more precise interpretation of the inflammatory heterogeneity of the disease. Finally, the consistency observed across independent layers of analysis reinforces the biological plausibility of our findings.

In conclusion, this study shows, using both clinical and immune data, that genetically confirmed HPP was associated with a high frequency of clinically diagnosed IMIDs in this referral cohort and with an altered systemic immune-inflammatory profile. Reduced ALP activity was associated with elevated inflammatory biomarkers, increased FCP, and coordinated alterations in leukocyte subpopulations, including diminished TNSALP expression and phenotypic changes consistent with altered systemic immune-inflammatory profile. These findings support a model in which TNSALP functions not only as a key regulator of bone mineralization but may also act as a modulator of immune homeostasis. In the context of HPP, impaired enzymatic activity may be associated with a sustained inflammatory environment and with immune-mediated comorbidities. Although confirmation in larger and longitudinal cohorts is still required, these data support further investigation of immune-inflammatory involvement in HPP. The cross-sectional design does not establish causality, and the exploratory models require external validation before any clinical application.

## Data Availability

The original contributions presented in the study are included in the article/**Supplementary Material**. Further inquiries can be directed to the corresponding authors.

## References

[1] McInnes IB Gravallese EM . Immune-mediated inflammatory disease therapeutics: past, present and future. Nat Rev Immunol. (2021) 21:680–6. doi: 10.1038/s41577-021-00603-1 34518662 PMC8436867

[2] El-Gabalawy H Guenther LC Bernstein CN . Epidemiology of immune-mediated inflammatory diseases: incidence, prevalence, natural history, and comorbidities. J Rheumatol Supplement. (2010) 85:2–10. doi: 10.3899/jrheum.091461 20436161

[3] Bezzio C Della Corte C Vernero M Di Luna I Manes G Saibeni S . Inflammatory bowel disease and immune-mediated inflammatory diseases: looking at the less frequent associations. Therap Adv Gastroenterol. (2022) 15:17562848221115312. doi: 10.1177/17562848221115312 35924080 PMC9340394

[4] Parlato M Charbit-Henrion F Pan J Romano C Duclaux-Loras R Le Du M-H . Human ALPI deficiency causes inflammatory bowel disease and highlights a key mechanism of gut homeostasis. EMBO Mol Med. (2018) 10:e8483. doi: 10.15252/emmm.201708483 29567797 PMC5887907

[5] Rader BA . Alkaline phosphatase, an unconventional immune protein. Front Immunol. (2017) 8:897. doi: 10.3389/fimmu.2017.00897 28824625 PMC5540973

[6] Pettengill M Matute JD Tresenriter M Hibbert J Burgner D Richmond P . Human alkaline phosphatase dephosphorylates microbial products and is elevated in preterm neonates with a history of late-onset sepsis. PloS One. (2017) 12:e0175936. doi: 10.1371/journal.pone.0175936 28448526 PMC5407836

[7] Sebastián-Serrano Á de Diego-García L Martínez-Frailes C Ávila J Zimmermann H Millán JL . Tissue-nonspecific alkaline phosphatase regulates purinergic transmission in the central nervous system during development and disease. Comput Struct Biotechnol J. (2014) 13:95–100. doi: 10.1016/j.csbj.2014.12.004 25709758 PMC4334957

[8] Bessueille L Briolay A Como J Mebarek S Mansouri C Gleizes M . Tissue-nonspecific alkaline phosphatase is an anti-inflammatory nucleotidase. Bone. (2020) 133:115262. doi: 10.1016/j.bone.2020.115262 32028019 PMC7185042

[9] Shanmugham LN Petrarca C Castellani ML Symeonidou I Frydas S Vecchiet J . IL-1beta induces alkaline phosphatase in human phagocytes. Arch Med Res. (2007) 38:39–44. doi: 10.1016/j.arcmed.2006.05.016 17174721

[10] Hernández-Chirlaque C Gámez-Belmonte R Ocón B Martínez-Moya P Wirtz S Sánchez de Medina F . Tissue non-specific alkaline phosphatase expression is needed for the full stimulation of T cells and T cell-dependent colitis. J Crohns Colitis. (2017) 11:857–70. doi: 10.1093/ecco-jcc/jjw222 28039309

[11] Mornet E . Hypophosphatasia. Metab Clin Exp. (2018) 82:142–55. doi: 10.1016/j.metabol.2017.08.013 28939177

[12] Mornet E Taillandier A Domingues C Dufour A Benaloun E Lavaud N . Hypophosphatasia: a genetic-based nosology and new insights in genotype-phenotype correlation. Eur J Hum Genet. (2021) 29:289–99. doi: 10.1038/s41431-020-00732-6 32973344 PMC7868366

[13] González-Cejudo T Villa-Suárez JM Ferrer-Millán M Andújar-Vera F Contreras-Bolívar V Andreo-López MC . Mild hypophosphatasia may be twice as prevalent as previously estimated: an effective clinical algorithm to detect undiagnosed cases. Clin Chem Lab Med (CCLM). (2023) 62 (1):128–37. doi: 10.1515/cclm-2023-0427 37440753

[14] García-Fontana C Villa-Suárez JM Andújar-Vera F González-Salvatierra S Martínez-Navajas G Real PJ . Epidemiological, clinical and genetic study of hypophosphatasia in a Spanish population: identification of two novel mutations in the Alpl gene. Sci Rep. (2019) 9:9569. doi: 10.1038/s41598-019-46004-2 31267001 PMC6606844

[15] Whyte MP . Hypophosphatasia - aetiology, nosology, pathogenesis, diagnosis and treatment. Nat Rev Endocrinol. (2016) 12:233–46. doi: 10.1038/nrendo.2016.14 26893260

[16] Akboğa F Hindilerden F Gültürk E Yönal Hindilerden İ Yılmaz A Deniz G . Increased perforin- and IL-21-expressing NK cells in patients with early-stage chronic lymphocytic leukemia. Experimed. (2022) 12:225–31. doi: 10.26650/experimed.1191622

[17] Patel KP Erickson AR Hearth-Holmes M . Systemic lupus erythematosus superimposed upon rare diagnosis of hypophosphatasia. JCR: J Clin Rheumatol. (2021) 27:S789. doi: 10.1097/RHU.0000000000001474 32649402

[18] Rauf MA Kotecha J Moss K . Reducing diagnostic delay in hypophosphatasia: a case series of 14 patients presenting to general rheumatology. Osteoporos Int. (2023) 34:1647–52. doi: 10.1007/s00198-023-06749-z 37118032 PMC10427523

[19] Shen NW Yi LG Omesiete W Peroutka CM Raghavan SS Greer KE . Cutaneous lesions in the setting of hypophosphatasia. JAAD Case Rep. (2023) 42:23–5. doi: 10.1016/j.jdcr.2023.10.001 37965189 PMC10641551

[20] Rodziewicz MS Moss K . 79. Hypophosphatasia presenting as seronegative arthritis and mistaken for psoriatic arthritis. Rheumatol Adv Pract. (2018) 2:rky034.042. doi: 10.1093/rap/rky034.042

[21] Alhumaidi H Meek S Malik A . MON-494 infantile hypophosphatasia diagnosed in adulthood: a case report. J Endocr Soc. (2019) 3:MON–494. doi: 10.1210/js.2019-MON-49431642201

[22] Girschick HJ Mornet E Beer M Warmuth-Metz M Schneider P . Chronic multifocal non-bacterial osteomyelitis in hypophosphatasia mimicking Malignancy. BMC Pediatr. (2007) 7:3. doi: 10.1186/1471-2431-7-3 17241478 PMC1784091

[23] Fertrin KY . Diagnosis and management of iron deficiency in chronic inflammatory conditions (CIC): is too little iron making your patient sick? Hematol Am Soc Hematol Educ Program. (2020) 2020:478–86. doi: 10.1182/hematology.2020000132 33275757 PMC7727593

[24] Ganz T . Anemia of inflammation. N Engl J Med. (2019) 381:1148–57. doi: 10.1056/NEJMra1804281 31532961

[25] Li H Zhao Y Li W Yang J Wu H . Critical role of neutrophil alkaline phosphatase in the antimicrobial function of neutrophils. Life Sci. (2016) 157:152–7. doi: 10.1016/j.lfs.2016.06.005 27287680

[26] Hossain A Jung LK . Expression of bone specific alkaline phosphatase on human B cells (86.18). J Immunol. (2007) 178:S127. doi: 10.4049/jimmunol.178.Supp.86.18 18579124

[27] Rambaldi A Terao M Bettoni S Tini ML Bassan R Barbui T . Expression of leukocyte alkaline phosphatase gene in normal and leukemic cells: regulation of the transcript by granulocyte colony-stimulating factor. Blood. (1990) 76:2565–71. doi: 10.1182/blood.V76.12.2565.2565 1702329

[28] Chen Y Xu Z Sun H Ouyang X Han Y Yu H . Regulation of CD8+ T memory and exhaustion by the mTOR signals. Cell Mol Immunol. (2023) 20:1023–39. doi: 10.1038/s41423-023-01064-3 37582972 PMC10468538

[29] Richter FC Saliutina M Hegazy AN Bergthaler A . Take my breath away—mitochondrial dysfunction drives CD8+ T cell exhaustion. Genes Immun. (2024) 25:4–6. doi: 10.1038/s41435-023-00233-8 38253749 PMC10873195

[30] Liu H Rhodes M Wiest DL Vignali DAA . On the dynamics of TCR:CD3 complex cell surface expression and downmodulation. Immunity. (2000) 13:665–75. doi: 10.1016/S1074-7613(00)00066-2 11114379

[31] Favier B Burroughs NJ Wedderburn L Valitutti S . TCR dynamics on the surface of living T cells. Int Immunol. (2001) 13:1525–32. doi: 10.1093/intimm/13.12.1525 11717193

[32] Ziegler-Heitbrock L Ancuta P Crowe S Dalod M Grau V Hart DN . Nomenclature of monocytes and dendritic cells in blood. Blood. (2010) 116:e74–80. doi: 10.1182/blood-2010-02-258558 20628149

[33] Radwan WM Khalifa KA Esaily HA Lashin NA . CD14++CD16+ monocyte subset expansion in rheumatoid arthritis patients: relation to disease activity and interleukin-17. Egyptian Rheumatol. (2016) 38:161–9. doi: 10.1016/j.ejr.2015.12.00242574925

[34] Koch S Kucharzik T Heidemann J Nusrat A Luegering A . Investigating the role of proinflammatory CD16+ monocytes in the pathogenesis of inflammatory bowel disease. Clin Exp Immunol. (2010) 161:332–41. doi: 10.1111/j.1365-2249.2010.04177.x 20456413 PMC2909416

[35] Sponaas AM Moen SH Liabakk NB Feyzi E Holien T Kvam S . The proportion of CD16+CD14dim monocytes increases with tumor cell load in bone marrow of patients with multiple myeloma. Immun Inflammation Dis. (2015) 3:94–102. doi: 10.1002/iid3.53 26029369 PMC4444152

[36] Strauss-Ayali D Conrad SM Mosser DM . Monocyte subpopulations and their differentiation patterns during infection. J Leukoc Biol. (2007) 82:244–52. doi: 10.1189/jlb.0307191 17475785

[37] Morrissey SM Geller AE Hu X Tieri D Ding C Klaes CK . A specific low-density neutrophil population correlates with hypercoagulation and disease severity in hospitalized COVID-19 patients. JCI Insight. (2021) 6:e148435. doi: 10.1172/jci.insight.148435 33986193 PMC8262329

[38] Opasawatchai A Amornsupawat P Jiravejchakul N Chan-in W Spoerk NJ Manopwisedjaroen K . Neutrophil activation and early features of NET formation are associated with dengue virus infection in human. Front Immunol. (2019) 9. doi: 10.3389/fimmu.2018.03007 30687301 PMC6336714

[39] Land WG . Role of DAMPs and cell death in autoimmune diseases: the example of multiple sclerosis. Genes Immun. (2023) 24:57–70. doi: 10.1038/s41435-023-00198-8 36750753

[40] Mansilla MJ Comabella M Río J Castilló J Castillo M Martin R . Up-regulation of inducible heat shock protein-70 expression in multiple sclerosis patients. Autoimmunity. (2014) 47:127–33. doi: 10.3109/08916934.2013.866104 24328534

[41] Shi J Xiao Y Zhang N Jiao M Tang X Dai C . HMGB1 from astrocytes promotes EAE by influencing the immune cell infiltration-associated functions of BMECs in mice. Neurosci Bull. (2022) 38:1303–14. doi: 10.1007/s12264-022-00890-1 35697993 PMC9672173

[42] Feldbush TL Lafrenz D . Alkaline phosphatase on activated B cells characterization of the expression of alkaline phosphatase on activated B cells. Kinetics and membrane anchor. J Immunol. (1991) 147:3690–5. doi: 10.4049/jimmunol.147.11.3690 1658149

[43] Burg DL Feldbush TL . Late events in B cell activation. Expression of membrane alkaline phosphatase activity. J Immunol. (1989) 142:381–7. doi: 10.4049/jimmunol.142.2.381 2783436

