# Supplementary material for: Tissue-nonspecific alkaline phosphatase deficiency is associated with altered immune cell profiles in hypophosphatasia
Source: Front Immunol. 2026 Sep 14;17:1906557. doi: 10.3389/fimmu.2026.1906557 (PMC13617094; doi:10.3389/fimmu.2026.1906557)
Supplement: Supplementary file 1 [file SupplementaryFile1.docx]

**SUPPLEMENTARY MATERIAL**

**Supplementary Table S1.** Lymphoid flow cytometry panel

| Marker | Fluorochrome | Clone |
| --- | --- | --- |
| TNSALP | BV421 | B4-78 |
| Mouse IgG1, k (Isotype Control) | BV421 | X40 |
| CD3 | BV510 | OKT3 |
| CD4 | FITC | RPA-T4 |
| CD19 | FITC | HIB19 |
| CD8 | APC | SK1 |

**Supplementary Table S2.** Myeloid flow cytometry panel

| Marker | Fluorochrome | Clone |
| --- | --- | --- |
| TNSALP | BV421 | B4-78 |
| Mouse IgG1, k (Isotype Control) | BV421 | X40 |
| CD14 | FITC | M5E2 |
| CD16 | PE | 3G8 |
| CD66b | APC | QA17A51 |

**Supplementary Table S3.** Clinical and biochemical parameters and genetic results of the study population.

| **Patient** | **Age** | **Sex** | **Mutation (DNA)** | **Protein consequence** | **NSAIDs** | **Glucocorticoid therapy** | **BMI (Kg/m^2^)** | **ALP (IU/L)** | **BALP (ng/mL)** | **FCP (µg/g)** | **Bone FX** | **Dental Loss** | **IMIDs** |
| --- | --- | --- | --- | --- | --- | --- | --- | --- | --- | --- | --- | --- | --- |
| P1 | 20 | M | c.17T>C | p.Leu6Ser | - | - | 25.4 | 40 | 7.8 | 29 | - | - | - |
| P2 | 21 | M | c.498_500delCAC | p.Thr167del | YES | - | 24.3 | 37* | 8.6 | 1213* | - | - | Crohn's Disease, Psoriasis |
| P3 | 67 | M | c.875C>T | p.Pro292Leu | - | - | 37.9 | 33* | - | 2301* | - | - | - |
| P4 | 37 | M | c.382G>A | p.Val128Met | - | - | 33 | 28* | 4.45* | 29 | - | - | - |
| P5 | 56 | M | c.571G>A | p.Glu191Lys | YES | - | 29.8 | 38* | 6.8 | 219* | - | - | - |
| P6 | 38 | M | c.1327G>A | p.Ala443Thr | - | YES | 31.5 | 29* | 2* | 94* | - | YES | psoriatic arthritis |
| P7 | 68 | F | c.473-2A>G; | Splicing donor | YES | YES | 33.9 | 23* | 2.9* | 122* | YES | YES | Poliarthritis, Osteochondritis, Synovitis, Acne, Pustulosis, Hyperostosis, Osteitis (SAPHO) |
| P8 | 47 | F | c.558G>A | p.Trp186* | - | - | 18.4 | 22* | 1.5* | 125* | - | - | Lymphocytic colitis |
| P9 | 35 | F | c.334G>C | p.Gly112Arg | - | - | 22.6 | 17* | 3.8* | 125* | - | - | Idiopathic arthritis |
| P10 | 72 | F | c.1328C>T | p.Ala443Val | YES | - | 29.9 | 23* | 4.05* | 64* | - | YES | - |
| P11 | 70 | M | c.382G>A | p.Val128Met | - | - | 29.7 | 28* | - | 51* | - | YES | - |
| P12 | 64 | F | c.493A>C | p.Thr165Trp | - | YES | 28.8 | 28* | 4.2* | 20 | - | YES | - |
| P13 | 57 | F | c.659G>T | p.Gly220Val | YES | - | 22.4 | 32* | 10.6 | 102* | YES | YES | Multiple sclerosis |
| P14 | 37 | F | C.558G>A | p.Trp186* | YES | - | 21 | 17* | 3.9* | 46 | - | YES | Ankylosing spondylitis |
| P15 | 38 | F | c.571G>A | p.Glu191Lys | YES | - | 28.4 | 24* | 3.55* | 62* | - | YES | - |
| P16 | 29 | F | c.352C>A | p.Leu118Met | - | - | 24.3 | 27* | 7 | 49 | - | - | - |
| P17 | 60 | F | c.1366G>A | p.Gly456Arg | YES | - | 35.4 | 20* | 2.6* | 1057* | YES | YES | - |
| P18 | 83 | F | c.473-2A>G | Splicing donor | - | - | 26.3 | 21* | 1.75* | 329* | YES | YES | - |
| P19 | 42 | F | c.558G>A | p.Trp186* | - | YES | 22.1 | 23* | 3.7* | 54* | YES | - | Immune thrombocytopenic purpura (ITP) |
| P20 | 61 | M | c.558G>A | p.Trp186* | YES | - | 34.4 | 39* | 6.1 | 59* | YES | YES | Chronic Atrophic Gastritis |
| P21 | 50 | M | c.659G>T | p.Gly220Val | - | - | 24.2 | 37* | 6.5 | 367* | - | YES | - |
| P22 | 48 | M | c.659G>T | p.Gly220Val | - | - | 25.4 | 47 | 9.7 | 26 | YES | YES | - |
| P23 | 52 | M | c.659G>T | p.Gly220Val | - | - | 28.9 | 33* | 4.7* | 30 | YES | - | - |
| P24 | 77 | F | c.1366G>A | p.Gly456Arg | YES | - | 24 | 21* | 2.4* | 2843* | YES | YES | Sjögren's síndrome |
| P25 | 61 | M | c.473-2A>G | Splicing donor | - | - | 29.4 | 26* | 4.8* | 30 | YES | - | Atopic dermatitis |
| P26 | 49 | F | c.1135C>A | p.His379Asn | - | - | 29.1 | 11* | - | 29 | YES | YES | - |
| P27 | 19 | M | c.1135C>A | p.His379Asn | - | - | 23.5 | 37* | 4.5* | 37 | YES | YES | - |
| P28 | 18 | F | c.1135C>A | p.His379Asn | - | - | 21 | 33* | 8 | 1 | - | YES | - |
| P29 | 78 | F | c.1327G>A | p.Ala443Thr | YES | - | 25.1 | 27* | 4.6* | 1413* | YES | YES | Chronic Atrophic Gastritis |
| P30 | 64 | M | c.1366G>A | p.Gly456Arg | - | - | 37.3 | 28* | - | - | - | YES | - |
| P31 | 34 | M | c.931G>A | p.Glu311Lys | - | - | 33.8 | 32* | 5.1* | - | YES | - | Hidradenitis suppurativa |
| P32 | 49 | F | c.473-2A>G | Splicing donor | - | YES | 20.8 | 21* | - | - | - | YES | Systemic lupus erythematosus |
| P33 | 64 | F | c.571G>A | p.Glu191Lys | - | - | 26.6 | 14* | 3.2* | - | YES | YES | - |
| P34 | 43 | F | c.407G>A | p.Arg136His | YES | - | - | 28* | - | 30 | YES | YES | - |
| P35 | 54 | M | c.498_500delCAC | p.Thr167del | - | - | 31 | 28* | 5.1* | - | YES | YES | - |
| P36 | 46 | F | c.1366G>A | p.Gly456Arg | - | - | 17.6 | 18* | 3.1* | - | - | YES | Atopic dermatitis |
| P37 | 54 | F | c.17T>C | p.Leu6Ser | - | - | 22.9 | 38 | 9.65 | - | - | - | - |
| P38 | 50 | F | c.473-2A>G | Splicing donor | - | - | 25.6 | 33* | 4.1* | - | - | YES | - |
| P39 | 80 | F | c.17T>C | p.Leu6Ser | YES | - | 27.5 | 16* | 2.4* | 166* | - | YES | Giant Cell Arteritis |
| P40 | 78 | F | c.1327G>A | p.Ala443Thr | - | - | 24.4 | 25* | 4.25* | 117* | YES | YES | Chronic Atrophic Gastritis |
| P41 | 33 | F | c.69_74delGAAAGA | p.Glu23_Lys24del | YES | - | 23.3 | 24* | - | 189.4* | - | - | Hashimoto Disease |
| P42 | 50 | F | c.17T>C | p.Leu6Ser | YES | - | 34 | 27* | - | - | - | YES | Psoriatic Arthritis |
| P43 | 62 | F | c.493A>C | p.Thr165Trp | - | - | 34.1 | 29* | 5.5* | - | - | YES | Sjögren's síndrome |
| P44 | 29 | M | c.659G>T | p.Gly220Val | - | - | 23.8 | 41 | 8.4 | - | YES | - | - |
| P45 | 52 | F | c.17T>C | p.Leu6Ser | - | - | 22.4 | 27* | 4.7* | - | YES | - | Psoriatic Arthritis |
| P46 | 33 | M | c.334G>C | p.Gly112Arg | - | - | 21.8 | 21* | 4.2* | - | - | - | - |
| P47 | 68 | M | c.1189+2insCTGGCCCCCATGCTG | Splicing aceptor | - | - |  | 28* | 4* | - | - | - | Sjögren's síndrome |

BMI: Body mass index; ALP: Serum alkaline phosphatase activity, (reference values: Female: 33-105 IU/L, Males: 40-120 IU/L); BALP: Tissue non-specific alkaline phosphatase bone isoform (reference values: 5.7-24.7 ng/mL); FCP: fecal calprotectin (reference values: < 50 µg/g); IMIDs: Inflammatory immune-mediated diseases; FX: fractures (* Values out of range)

**Supplementary Table S4.** FCP and ALP quadratic polynomial regression

|  | Estimate | Std. Error | t value | Pr(>\|t\|) |
| --- | --- | --- | --- | --- |
| (Intercept) | 3.2498778 | 0.5259133 | 6.179 | 7.3e-08 *** |
| ALP (IU/L) | -0.0647617 | 0.0219136 | -2.955 | 0.00454 ** |
| I(ALP (IU/L)^2) | 0.0004817 | 0.0001835 | 2.625 | 0.01111 * |
| Adjusted R-squared: 0.1298 | | | | |
| F-statistic: 5.402 on 2 and 57 DF, p-value: 0.007107 | | | | |

(*) *p* < 0.05, (**) *p* < 0.01, (***) *p* < 0.001

**Supplementary Table S5.** CRP and ALP quadratic polynomial regression

|  | Estimate | Std. Error | t value | Pr(>\|t\|) |
| --- | --- | --- | --- | --- |
| (Intercept) | 7.543e-01 | 1.683e-01 | 4.482 | 3.32e-05 *** |
| ALP (IU/L) | -2.020e-02 | 7.299e-03 | -2.767 | 0.00747 ** |
| I(ALP (IU/L)^2) | 1.657e-04 | 6.173e-05 | 2.684 | 0.00935 ** |
| Adjusted R-squared: 0.08271 | | | | |
| F-statistic: 3.84 on 2 and 61 DF, *p*-value: 0.02686 | | | | |

(**) *p* < 0.01, (***) *p* < 0.001

**Supplementary Figure S1.** Lymphoid flow cytometry panel

**Supplementary Figure S2.** Myeloid flow cytometry panel

**Supplementary Table S6.** Exploratory Bayesian logistic regression analysis of IMID status according to ALP activity

| **Predictor** | **N** | **IMID events** | **Predictors** | **Events per variable (EPV)** | **OR** | **95% IC** | **Bayesian *p*-value** | **Apparent AUC** | **Estimated Optimism** | **Optimism-Corrected AUC** |
| --- | --- | --- | --- | --- | --- | --- | --- | --- | --- | --- |
| ALP (IU/L) | 47 | 22 | 1 | 22 | 0.925 | 0.846–0.999 | 0.025 | 0.681 | 0.005 | 0.671 |

**Supplementary Table S7.** Exploratory Bayesian logistic regression analysis of IMID status according to FCP levels

| **Predictor** | **N** | **IMID events** | **Predictors** | **EPV** | **OR** | **95% IC** | **Bayesian *p*-value** | **Apparent AUC** | **Estimated Optimism** | **Optimism-Corrected AUC** |
| --- | --- | --- | --- | --- | --- | --- | --- | --- | --- | --- |
| log10(FCP) | 33 | 15 | 1 | 15 | **5** | 2.54–9.99 | <0.001 | 0.717 | 0.001 | 0.707 |

**Supplementary Table S8.** Exploratory Bayesian logistic regression analysis of IMID status according to ALP activity and FCP levels

| **Predictor** | **N** | **IMID events** | **Predictors** | **EPV** | **OR** | **95% IC** | **Bayesian *p*-value** | **Apparent AUC** | **Estimated Optimism** | **Optimism-Corrected AUC** |
| --- | --- | --- | --- | --- | --- | --- | --- | --- | --- | --- |
| ALP ≥28 | 33 | 15 | 2 | 7.5 | 0.217 | 0.041–0.969 | 0.022 | 0.77 | 0.034 | 0.736 |
| log10(FCP) | 33 | 15 | 2 | 7.5 | 2.371 | 0.701–9.236 | 0.084 |  |  |  |

**Supplementary Table S9.** Exploratory LASSO logistic regression analysis of elevated fecal calprotectin status according to BALP, TSI, and age

| **Predictor** | **N** | **FCP > 50 µg/g events** | **Predictors** | **EPV** | **LASSO β** | **Apparent AUC** | **Estimated Optimism** | **Optimism-Corrected AUC** |
| --- | --- | --- | --- | --- | --- | --- | --- | --- |
| BALP (ng/mL) | 27 | 18 | 3 | 6 | −0.143 | 0.883 | 0.030 | 0.853 |
| TSI (%) | 27 | 18 | 3 | 6 | −0.137 |  |  |  |
| Age (years) | 27 | 18 | 3 | 6 | 0.018 |  |  |  |

**Supplementary Table S10.** Logistic regression analysis of IMID status according to CD8⁺ NK cells

| **Predictor** | **N** | **IMID events** | **Predictors** | **EPV** | **OR** | **95% CI** | ***p*-value** | **Apparent AUC** | **Estimated Optimism** | **Optimism-Corrected AUC** |
| --- | --- | --- | --- | --- | --- | --- | --- | --- | --- | --- |
| CD8⁺ NK-enriched cells (%) | 28 | 13 | 1 | 13 | 0.807 | 0.632–0.977 | 0.026 | 0.749 | -0.004 | 0.753 |

**Supplementary Table S11.** Logistic regression analysis of IMID status according to TNSALP⁺ B cells

| **Predictor** | **N** | **IMID events** | **Predictors** | **EPV** | **OR** | **95% CI** | ***p*-value** | **Apparent AUC** | **Estimated Optimism** | **Optimism-Corrected AUC** |
| --- | --- | --- | --- | --- | --- | --- | --- | --- | --- | --- |
| TNSALP⁺ B cells (%) | 28 | 13 | 1 | 13 | 0.587 | 0.301–0.973 | 0.037 | 0.726 | -0.007 | 0.733 |

**Supplementary Table S12.** Logistic regression analysis of IMID status according to immunophenotypic variables

| **Predictor** | **N** | **IMID events** | **Predictors** | **EPV** | **OR** | **95% CI** | ***p*-value** | **Apparent AUC** | **Estimated Optimism** | **Optimism-Corrected AUC** |
| --- | --- | --- | --- | --- | --- | --- | --- | --- | --- | --- |
| TNSALP⁺ B cells (%) | 28 | 13 | 4 | 3.25 | 0.601 | 0.301–0.965 | 0.034 | 0.856 | 0.086 | 0.77 |
| CD8⁺ NK-enriched cells (%) | 28 | 13 | 4 | 3.25 | 0.778 | 0.576–0.982 | 0.033 |  |  |  |
| Sex | 28 | 13 | 4 | 3.25 | 0.646 | 0.087–3.793 | 0.635 |  |  |  |
| Age (years) | 28 | 13 | 4 | 3.25 | 1.011 | 0.960–1.067 | 0.672 |  |  |  |
